# Interface Energy Coupling between *β*-tungsten Nanofilm and Few-layered Graphene

**DOI:** 10.1038/s41598-017-12389-1

**Published:** 2017-09-22

**Authors:** Meng Han, Pengyu Yuan, Jing Liu, Shuyao Si, Xiaolong Zhao, Yanan Yue, Xinwei Wang, Xiangheng Xiao

**Affiliations:** 1 Department of Mechanical Engineering, Iowa State University, 2010 Black Engineering Building, Ames, IA 50011 USA; 2 Department of Physics and Key Laboratory of Artificial Micro- and Nano-structures of Ministry of Education, Hubei Nuclear Solid Physics Key Laboratory and Center for Ion Beam Application, Wuhan University, Wuhan, 430072 P. R. China; 3 School of Power and Mechanical Engineering, Wuhan University, Wuhan, Hubei 430072 P. R. China

## Abstract

We report the thermal conductance induced by few-layered graphene (G) sandwiched between *β*-phase tungsten (*β*-W) films of 15, 30 and 40 nm thickness. Our differential characterization is able to distinguish the thermal conductance of *β*-W film and *β*-W/G interface. The cross-plane thermal conductivity (*k*) of *β*-W films is determined at 1.69~2.41 Wm^−1^K^−1^ which is much smaller than that of *α*-phase tungsten (174 Wm^−1^K^−1^). This small value is consistent with the large electrical resistivity reported for *β*-W in literatures and in this work. The *β*-W/*β*-W and *β*-W/G interface thermal conductance (*G*_*W/W*_ and *G*_*W/G*_) are characterized and compared using multilayered *β*-W films with and without sandwiched graphene layers. The average *G*_*W/W*_ is found to be at 280 MW m^−2^K^−1^. *G*_*W/G*_ features strong variation from sample to sample, and has a lower-limit of 84 MW m^−2^K^−1^, taking into consideration of the uncertainties. This is attributed to possible graphene structure damage and variation during graphene transfer and W sputtering. The difference between *G*_2*W/G*_ and *G*_*W/W*_ uncovers the finite thermal resistance induced by the graphene layer. Compared with up-to-date reported graphene interface thermal conductance, the *β*-W/G interface is at the high end in terms of local energy coupling.

## Introduction

Monolayered or few-layered graphene has attracted remarkable attention over the past several years due to its extremely high electron mobility and thermal conductivity^1–4^. Although there still remains challenges in wafer-scale deposition and controlling the electronic bandgap, graphene is widely seen as a strong candidate for future microelectronics^5–7^. In the applications of graphene, interface thermal resistance (*R*) or conductance (*G*) induced by graphene is the most common quantity used to characterize heat dissipation from graphene to its substrate. Early work by Freitage *et al*.^8^ characterized the heat dissipation from graphene to substrate for the first time, but didn’t explore graphene-substrate interface thermal resistance. Following work by Chen *et al*.^9^ employed the differential 3*ω* method on the graphene/SiO_2_ interface and reported a *R* range from 5.6 × 10^−9^ to 1.2 × 10^−8^ Km^2^W^−1^ at room temperature. Mak *et al*.^10^ employed the ultrafast pump-probe method and obtained a *G* of the single-layered and multilayered graphene/SiO_2_ interface varying from 2000 to 11000 Wcm^−2^K^−1^. Koh *et al*.^11^ performed the time-domain thermoreflectance (TDTR) measurement on the Au/Ti/graphene/SiO_2_ and Au/Ti/SiO_2_ sandwiches (graphene layers 1 ≤ *n* ≤ 10) and reported a *G* of ~25 MWm^−2^K^−1^ at room temperature for the Au/Ti/graphene/SiO_2_ interface. Similarly, Guzman *et al*.^12^ performed the TDTR measurements on the variable metals/single-layered graphene/SiO_2_ structures and gave a *G* range of 15-60 MWm^−2^K^−1^. Hopkins *et al*.^13^ determined the thermal boundary conductance across the Al/single-layered graphene/Si interface from the TDTR measurement and found a two-fold increase with the oxygen functionalization of the graphene.

Despite the extensive studies of metal/graphene/SiO_2_ interfaces, the metal/graphene/metal interfaces with metal substrates like Cu, Pd and W are lack of attention until recent years^14–16^. Huang *et al*.^16^ performed the TDTR measurements on the Pd/transferred graphene (trG)/Pd interface and reported a *G* of 300 MWm^−2^K^−1^ at room temperature for the sample with radiofrequency (rf) magnetron sputtering top Pd. This value is seven times larger than that with thermal evaporation top Pd (42 MWm^−2^K^−1^). The large enhancement of thermal conductance is attributed to the electronic heat conduction via atomic scale pinholes formed in the graphene during the sputtering process. W, as an excellent radiation tolerance material^17–19^, is of great importance for the safety of nuclear reactors. Graphene, because of its impermeability to all standard gases (including He)^20^, has also been regarded as an effective material in retarding radiation damages during nuclear reaction^21^. These remarkable properties of W and graphene motivate us to fabricate the W/graphene multilayered system and study the ability of its radiation tolerance and the resulting thermal transport capacity. As a special phase of W, *β*-W has been extensively investigated due to its high superconducting transition temperature^22–25^ since first prepared by Hartmann^26^ and Fink^27^. In recent years, investigations of *β*-W have mainly focused on the nanostructure, phase transformation conditions, residual stress, electrical resistivity and spin Hall effect^28–33^. No direct characterization of the thermal transport properties of this unique metastable structure has been reported.

In the present work, we systemically study the structure and thermal transport properties of the multilayered *β*-W films sandwiched with graphene layers by employing the photothermal (PT) technique^34–36^. In the PT technique, the sample is heated by an intensity-periodic laser and the thermal radiation signals from the sample surface are detected by an infrared detector. By fitting the phase shift between the radiation signals and the incident laser beam, the cross-plane thermal conductivity (*k*) of *β*-W, *β*-W/*β*-W interface thermal resistance (*R*_*W/W*_) and *β*-W/G interface thermal resistance (*R*_*W/G*_) are determined. Furthermore four-probe electrical resistivity study is carried out on the measured samples and compared with the PT measurement results to interpret the experimental observations.

## Results

### Thermal Conductivity of single-layered *β*-W

**Figure 1:**
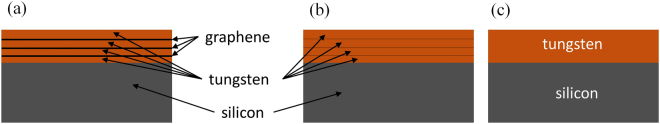
Schematic of the samples in our experiment. (**a)** Multilayered *β*-W sandwiched with graphene layers on 100-nm-SiO_2_/Si substrate (named A, B, D). (**b**) Multilayered *β*-W on 100-nm-SiO_2_/Si substrate (named a, b, d). (**c**) Single-layered *β*-W on 100-nm-SiO_2_/Si substrate (named Aa, Bb, Dd).

**Figure 2:**
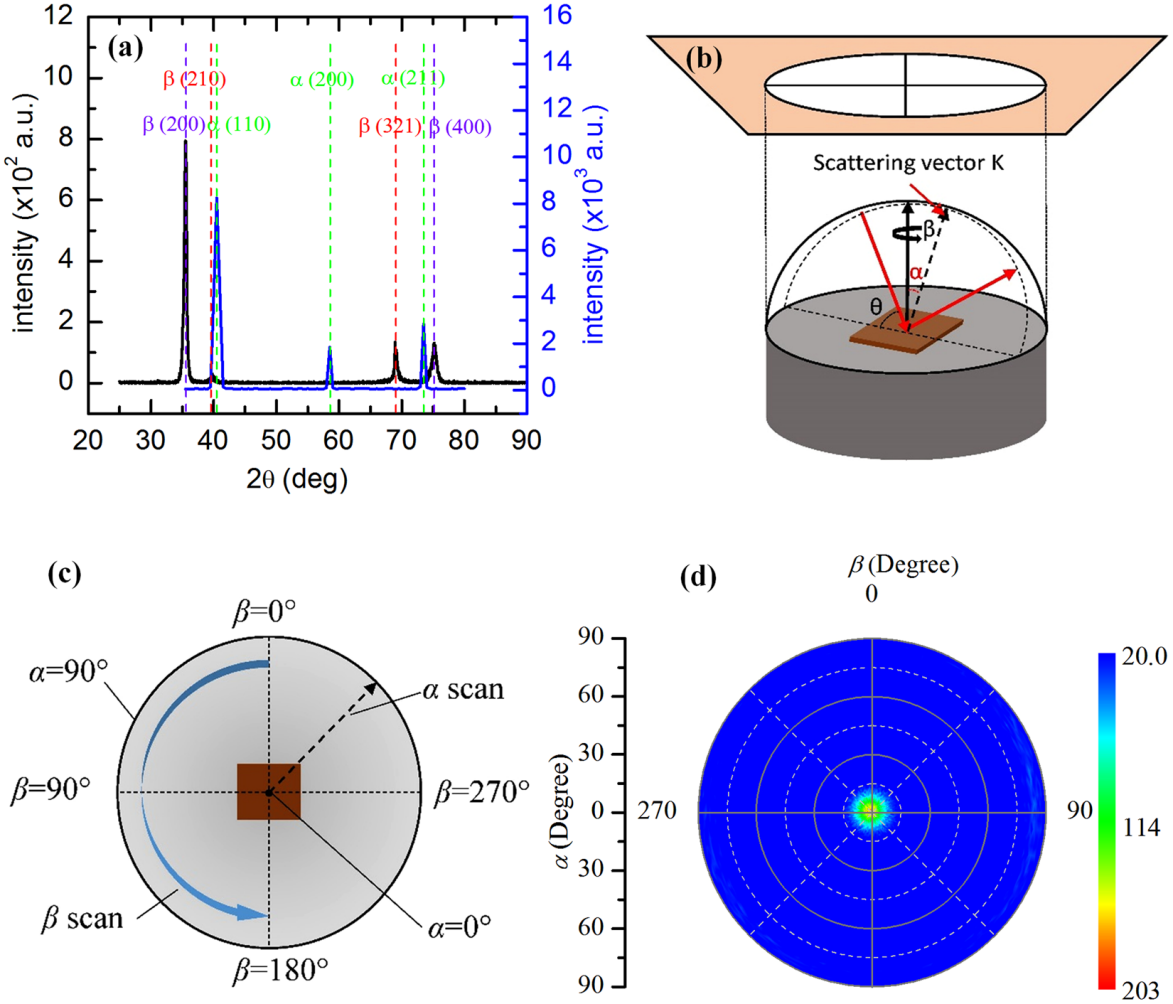
(**a**) XRD patterns. The black line is taken from one of our *β*-W films (sample Dd3: single-layered tungsten on 100-nm-SiO_2_/Si substrate with a thickness of 344 nm) and the blue line is from ref.^61^, which is *α*-W films coated on stainless steel. Due to the same sample preparation condition, the black line is a representative for all the samples in our experiment. This blue line serves as a comparison to show the differences of phase composition and crystal structure of tungsten films obtained under different conditions. (**b**) Sphere of fixed-length scattering vector and stereographic projection in pole-figure XRD. (**c**) Definition of *α* and *β* in the pole-figure measurement. (**d**) Pole-figure for *β*(200) plane (sample Dd3). The maximum pole lies in the center of the pole figure, which means that *β*(200) plane is parallel to the sample surface.

The single-layered W films on 100-nm-SiO_2_/Si substrate [see Fig. 1(c)] are prepared by using the vacuum magnetron sputtering system. Also shown in Fig. 1 are the structures of multilayered W films without and with sandwiched graphene layers. Detailed sample structure and thickness are listed in the Supplementary materials (see Table 1 in S1). W layer thicknesses are measured in the Digital Micrograph from the scanning electron microscopy (SEM) images that will be discussed later. Uncertainties may come from the aspects of SEM images and the measurement operation in the Digital Micrograph. Through x-ray diffraction (XRD) analysis [see Fig. 2(a,b,c)], the W films are determined to be *β*-W with the (200) plane parallel to the surface [see Fig. 2(d)]. Details of the sample preparation and structure characterization can be found in the Methods section.

**Figure 3:**
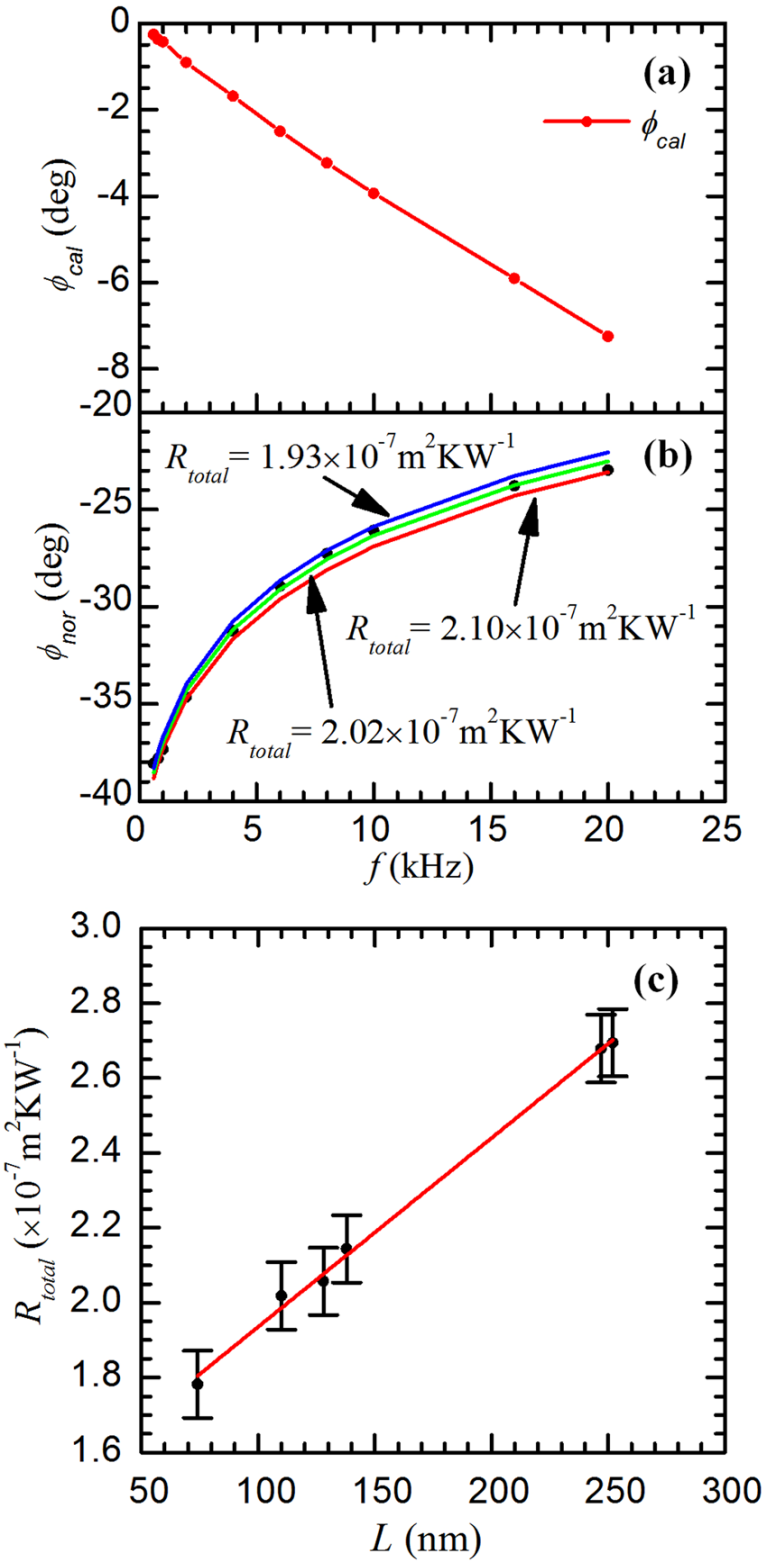
(**a**) Phase shift of the reflection beam that serves as a calibration of the experimental system. (**b**) Phase shift fitting result of sample Aa2 (single-layered *β*-W with a thickness of 110 nm). The total thermal resistance (*R*_*total*_) is determined at 2.02 × 10^−7^ Km^2^W^−1^ with the best fitting (green line) for this sample. The blue line and red line are theoretical curves when *R*_*total*_ takes the value of 1.93 × 10^−7^ Km^2^W^−1^ and 2.10 × 10^−7^ Km^2^W^−1^ to show the fitting sensitivity. (**c**) Linear fitting of *R*_*total*_ versus *L* from which the thermal conductivity *k* of *β*-W films and thermal resistance *R*_*W/Si*_ between *β*-W film and Si substrate are determined at 1.98 Wm^−1^K^−1^ and 1.43 × 10^−7^ Km^2^W^−1^, respectively.

In this work, the PT technique is used to characterize the thermal transport properties of the films. It irradiates the sample surface with an amplitude modulated laser beam, and measures the surface thermal radiation. The laser beam modulation frequency is scanned within a wide range, and the time delay (phase shift) between the thermal radiation and laser beam is measured and used for data fitting. The phase shift is related to the physical properties of the sample such as thermal conductivity, heat capacity and density of the *β*-W films as well as the thermal resistance induced by the SiO_2_ layer and the *β*-W/SiO_2_ interface. Given these physical properties, the theoretical phase shift can be calculated. The physical model of the PT technique and the experimental setup are detailed in the Supplementary materials (see S2). In the experimental measurement, we are interested in the phase shift between the thermal radiation and the modulated laser beam. However, the measurement will inevitably include time delay induced by the whole system. This time delay can be eliminated by measuring the phase shift between the reflected laser beam and the irradiating laser beam (noted as *ϕ*_*cal*_). The experimental setup for the calibration process has no other difference except for detecting the reflected laser signals instead of the thermal radiation by removing the Ge window. Figure 3(a) shows the phase shift of the reflected laser beam. The system phase shift shows a straight line against the modulation frequency, indicating a constant time delay of the system within the experiment frequency range. This time delay is estimated to be around 1.0 × 10^−6^ s. The real experimental phase shift (noted as *ϕ*_*nor*_) between the thermal radiation and the laser beam can be calculated as *ϕ*_*nor*_ = *ϕ*_*raw*_ − *ϕ*_*cal*_, with *ϕ*_*raw*_ being the raw phase shift detected in this experiment.

The fitting process is operated by using a well-developed program by our lab. By using different trail values of unknown parameters, the theoretical phase shifts are calculated over the specified modulation frequency range. The value that gives the least square deviation between the theoretical phase shifts and the experimental ones is taken as the real property of materials. Here, the thermal resistance induced by the SiO_2_ layer and the *β*-W/SiO_2_ interface (noted as ${R}_{W/Si{O}_{2}/Si}$) and the cross-plane *k* of *β*-W films are both unknown. However, one single measurement of the sample cannot distinguish these two properties. What we can get from the fitting of one sample measurement is the total thermal resistance (*R*_*total*_) of the sample, which includes both the thermal resistance induced by the *β*-W film and ${R}_{W/Si{O}_{2}/Si}$. Then we measure samples of different thickness that are synthesized under the exactly same conditions to vary the effect of thermal resistance of the *β*-W film. By studying how *R*_*total*_ varies with the W film thickness (*L*), *k* of *β*-W film and ${R}_{W/Si{O}_{2}/Si}$ can be distinguished and determined.

Figure 3 (b) shows the fitting result for sample Aa2 to demonstrate the fitting agreement extension. This is a *β*-W film of 110 nm thickness. Excellent agreement is observed between the fitting (green line) and experimental data (black circles) at all the modulation frequencies with a fitting residue of 0.30 degree. *R*_*total*_ is determined to be 2.02 × 10^−7^ Km^2^W^−1^ for this sample. When *R*_*total*_ is taken as 1.93 × 10^−7^ Km^2^W^−1^ and 2.10 × 10^−7^ Km^2^W^−1^, the fitting residue is 0.53 and 0.51, respectively, which are much larger than the experimental uncertainty of the phase shift as shown in Fig. 3(b). The theoretical fitting curves of these values (blue line and the red one) also show obvious deviation from the best fitting curve (the green one) in Fig. 3(b). Therefore, the uncertainty of the measured thermal resistance is +0.08/−0.09 × 10^−7^ Km^2^W^−1^.

*R*_*total*_ of the samples can be expressed as the following equation:

1 $${R}_{total}=L/k+{R}_{W/Si{O}_{2}/Si},$$

Since all the *β*-W films are prepared using the same substrate and under the same conditions, *k* of *β*-W films and ${R}_{W/Si{O}_{2}/Si}$ are expected to have negligible variation among samples (despite the film size effect which will be discussed later). As a result, we expect a linear relation between *R*_*total*_ and *L*. Figure 3(c) shows the measured *R*_*total*_ versus *L* for the single-layered *β*-W films studied in this work. An obvious linear relation is observed. Based on linear fitting, *k* of *β*-W films and ${R}_{W/Si{O}_{2}/Si}$ are determined at (1.98 ± 0.06) Wm^−1^K^−1^ and (1.43 ± 0.02) × 10^−7^ Km^2^W^−1^, respectively. In the range of the laser modulation frequency (600 Hz to 20 kHz), the thermal diffusion depth in one period ($\sqrt{\alpha /f}$) varies from 35 μm to 6.1 μm, which is much larger than the sample thickness. However, thermal diffusion depth in one-degree phase ($\sqrt{\alpha /f\cdot 360}$) varies from 1.9 μm to 0.32 μm, which is comparable with the thickness of the sample. Figure 3(b) also shows that the phase shift method is sensitive in our experiment. The *k* value is much smaller than that of bulk *α*-W (174 Wm^−1^K^−1^). As there is no record for *k* of *β*-W, we will try to explain this low thermal conductivity of the metastable *β*-W from the Wiedemann-Franz (WF) law assuming that electrons still dominate in thermal conductance of this material.

Thermal conductivity is related to the electrical conductivity (*σ*) by the WF law at relatively not-very-low temperatures by the following equation:

2 $${L}_{lorenz}=\frac{k}{\sigma T},$$

where *L*_*Lorenz*_ is the Lorenz number and for tungsten *L*_*Lorenz*_ is 3.04 × 10^−8^ W·ΩK^−2^ at 300 K^37^. *σ* is calculated at 2.17 × 10^5^ Ω^−1^m^−1^ based on our measured *k*, corresponding to an electrical resistivity of 4.61 × 10^−6^ Ω·m (or 461 µΩ·cm). This value is comparable with the reported electrical resistivity for this phase of W^29–32,38,39^. Early work by Petroff *et al*.^38^ reported an electrical resistivity of *β*-W ranges from 150–350 µΩ·cm. Following work by O’Keefe *et al*.^30^ reported an even higher electrical resistivity of as-deposited *β*-W (155–870 µΩ·cm) and found that even after rapid thermal annealing, it can still be as high as 478 µΩ·cm. Recent work by Hao *et al*.^32^ measured the electrical resistivity of *β*-W with different thicknesses and extracted a value of about 195 µΩ·cm. The relatively high electrical resistivity (compared with 5.33 µΩ·cm for the bulk pure *α*-W^32^) will be discussed in detail below.

As the film is very thin, the cross-plane *σ* is not easy to measure directly. We have measured the in-plane *σ* of the films by using the four-probe method for comparison^40,41^. Details of the experiment setup of the four-probe method can be found in the Supplementary materials (see S3). A current *I* is fed through the two outer probes and the voltage *V* is measured between the two inner ones. The sheet resistivity of samples can be expressed as: *R*_*s*_ *= F*_1_ *F*_2_ *F*_3_ *V/I*, where *F*_1_ is the finite shape correction factor, *F*_2_ the probe spacing correction factor and *F*_3_ the thickness correction factor. *F*_1_ can be derived from the existing standard table^42^. *F*_2_ can be calculated by the following equation^42^:

3 $${F}_{2}=1+1.082\cdot (1-\frac{{S}_{2}}{S}),$$

where *S*_2_ is the spacing between the two inner probes and *S* the average probes spacing. In our experiment, with equal spacing between adjacent probes, *F*_*2*_ is taken as 1. *F*_3_ is also taken as 1 for all the samples, as in our case the thickness (*t*) of all these films is very small which means *t/s* << 0.4.

**Figure 4:**
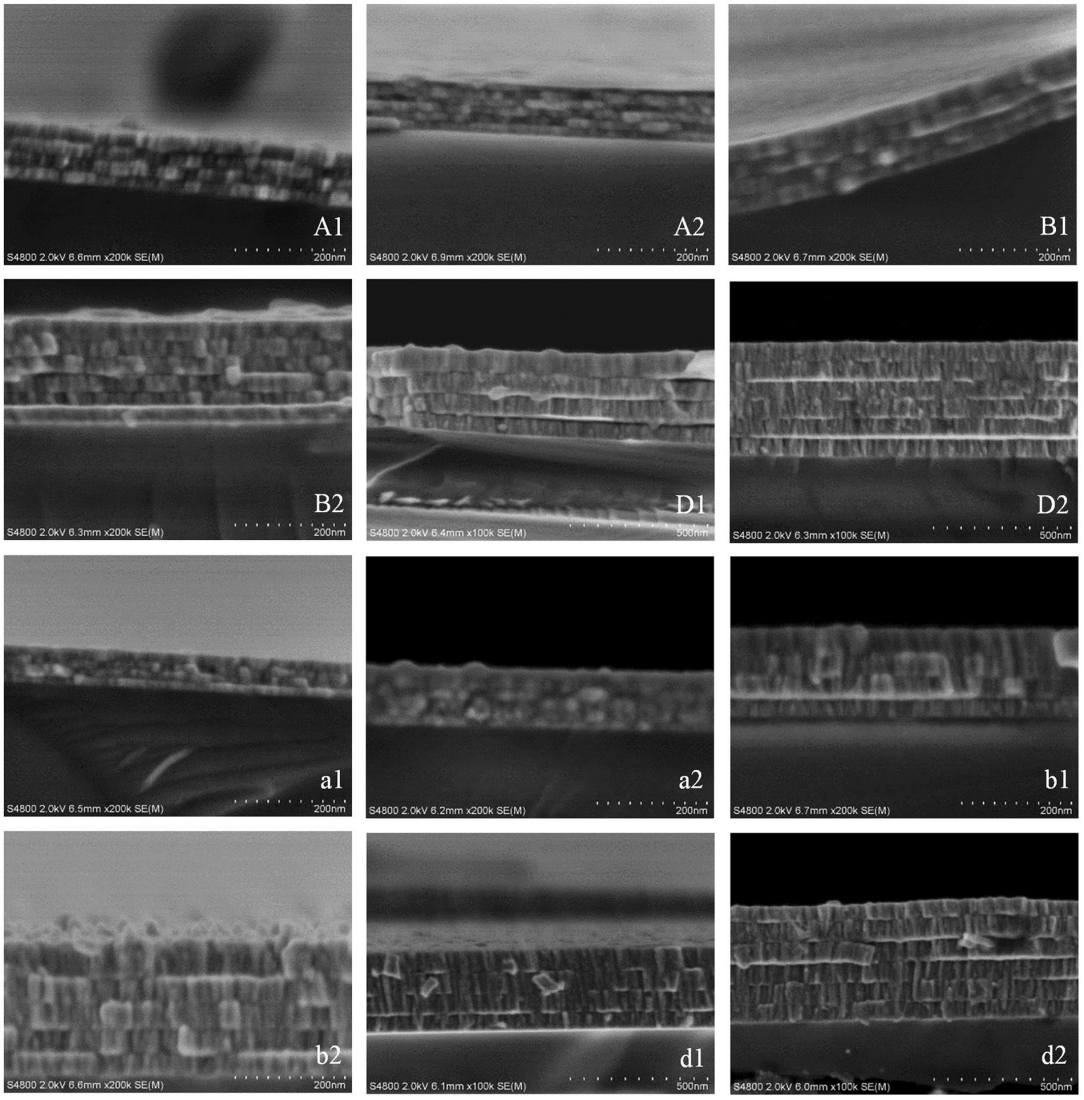
SEM image of the multilayered *β*-W films from which we can see the clear interface between sublayers. The layer thickness is directly measured based on the SEM image.

In-plane *σ* of *β*-W is calculated as $\sigma =1/({R}_{s}t)$ and the results can be found in the Supplementary materials (see Table 2 in S4). The measured in-plane *electrical resistivity σ* ^−1^ varies from 258 to 316 µΩ·cm which is in the well accepted range (150–350 µΩ·cm) of *β*-W^38^. Typically, the relatively higher resistivity in thin metallic films is attributed to the carrier momentum loss along the current flow direction due to surface and grain boundary scattering which is commonly explained by using the Fuchs-Sondhermer (FS) surface scattering model^43,44^ and the Mayadas-Shatzkes (MS) grain boundary model^45,46^, respectively. However, the finite-size effect and the grain boundary scattering are not sufficient to account for the large resistivity of *β*-W. According to Hao’s recent work, the electrical resistivity of bulk *β*-W is calculated at 195 ± 3 µΩ·cm with an effective mean free path (*l*_*eff*_) of about only 0.45 nm at 300 K^32^. This means *l*_*eff*_ of *β*-W films may be ten to hundred times smaller than the averaged grain size and the film thickness [see Fig. 4] of our samples. Calculations using the FS model and the MS model show that when the thickness or grain size is much larger than *l*_*eff*_, surface scattering or grain boundary scattering has negligible influence on the resistivity of thin metallic films^44^. Other scattering mechanism must exist and dominate the electron transport property. One possible explanation is the charge carrier concentration. Recent work by Lee *et al*.^39^ obtained the charge carrier concentration of different phase of W by using Hall measurement and found a much low carrier concentration of the *β*-phase one. Another possible reason is the impurities, where a small amount of oxygen is believed to exist and induce the *β*-W formation without forming a W_x_O compound^47^. Such a dilute bulk of impurities may induce significant electron scattering. This also helps explain the large range of reported resistivity of *β*-W which may be due to the different concentration of oxygen induced impurities. The local structure may be another reason as *β*-W is believed to be a mixed phase consisting of ordered and stacking faulted W_3_W structures^38^. Considering the relatively larger grain size, local disorders or dislocations of atoms may also play an important role. So far, as there is no clear mechanism for the large resistivity, further work, particularly theoretical study, is needed.

The discrepancy in the thermal conductivity determined by the PT technique and the four-probe method is mainly attributed to the anisotropy of the *β*-W films. The *β*-W films are produced in the vacuum magnetron sputtering system, where the deposited metal films typically show kind of columnar structure. For the tungsten films prepared in this work, the columnar structure can be clearly seen from the SEM images in Fig. 4. This means the crystal orientation and crystallite size in the in-plane and cross-plane directions are much different. Thus, the electrical and thermal transport properties which are closely related to the structure of materials also have much difference in the two directions. Despite this anisotropy in *σ* and *k*, the validation of WF law is also questionable. It has been widely studied that, for nanocrystalline films, the Lorenz numbers are very different from their corresponding bulk values. For example, Yoneoka *et al*.^48^ reported an average Lorenz number of 3.82 × 10^−8^, 2.79 × 10^−8^, and 2.99 × 10^−8^ WΩK^−2^ for 7.3-, 9.8-, and 12.1-nm Pt films, respectively. Experimental results of Zhang and co-workers^49,50^ showed that the Lorenz number of 21–37 nm and 53 nm thick polycrystalline Au films are around 7.0 × 10^−8^ and 5.0 × 10^−8^ WΩK^−2^, respectively. Calculations by Ou *et al*.^51^ revealed that the Lorenz number of a 180-nm nickel nanowire is a little higher than the bulk value. Our previous experimental work^52^ on ultrathin iridium films (0.6–7 nm) shows that the Lorenz number ranges from 5.83 × 10^−8^ to 7.8 × 10^−8^ WΩK^−2^. As there is no literature data for the Lorenz number of *β*-W nanofilms and how it will deviate from the bulk value is not clear to our knowledge, the use of WF law based on the bulk’s Lorenz number is not suitable in this work. Therefore, considering the unclear anisotropy level of the *β*-W nanofilms and the deviation of the Lorenz number, we intend to extract cross-plane direction *k* directly by performing the PT measurements on this group of samples in this section.

### Thermal conductance between *β*-W sublayers

**Figure 5:**
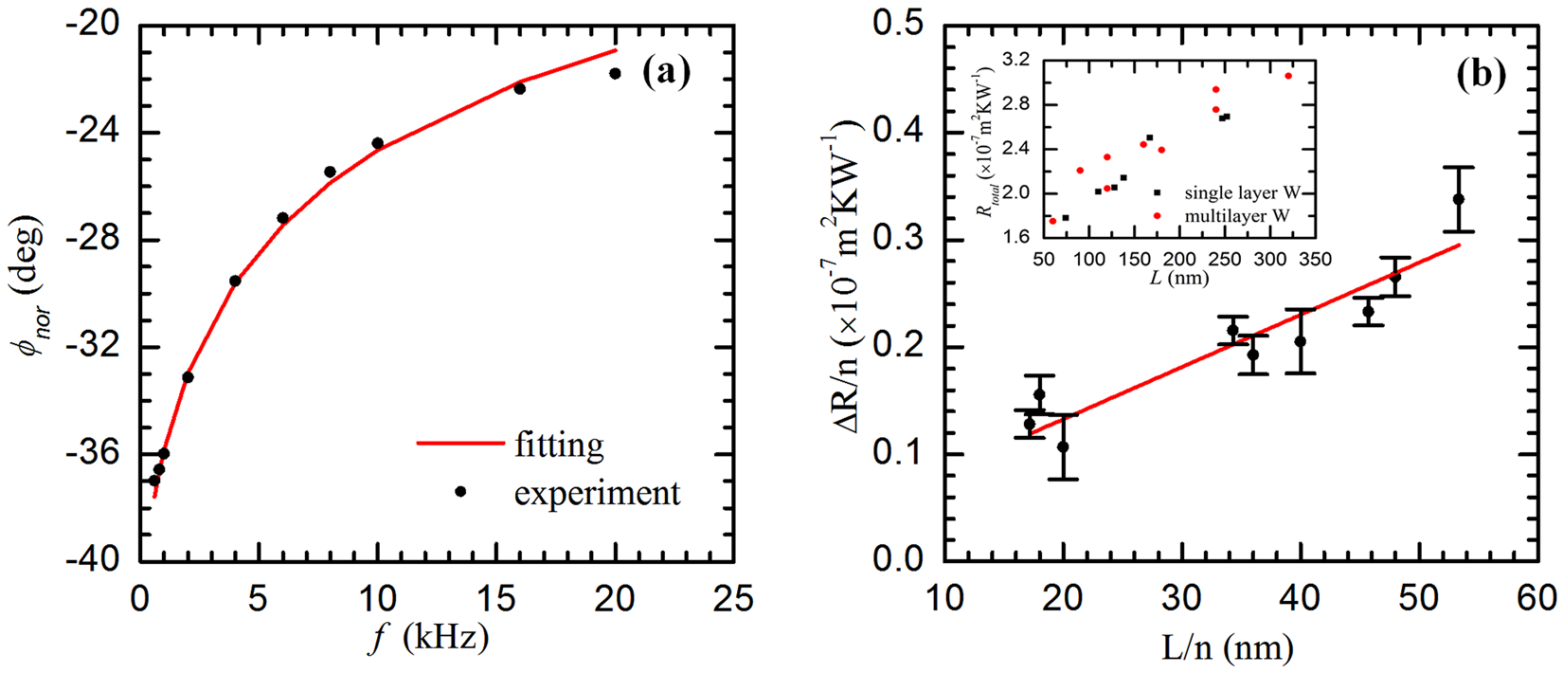
(**a**) Phase shift fitting of sample a3 (8 layers of *β*-W films with a thickness of 120 nm in total). The total thermal resistance (*R*_*total*_) is determined at 2.05 × 10^−7^ Km^2^W^−1^ for this sample. (**b**) Linear fitting of Δ*R/n* versus *L/n*, from which the thermal resistance *R*_*W/W*_ between *β*-W sublayers is determined at 3.57 × 10^−9^ Km^2^W^−1^. The inset shows total thermal resistance versus sample thickness of both the single-layered (black rectangles) and the multilayered (red circles) *β*-W films.

Not like the samples in the first group, the samples in this group were grown for several times, that is, these samples consist of different number of sublayers [see Fig. 1(b)]. The SEM images also clearly show separated layers in these films [see Fig. 4]. The experiments are operated under the same conditions as those of the first group. Notice that, in the fitting process of the first group for a single-layered sample, the fitting itself cannot distinguish the thermal resistance of the *β*-W films from other resistances. This also holds true for the multilayered *β*-W samples. Figure 5(a) shows the fitting process, from which we can see the fitting curve matches the experimental data well. After the fitting process, *R*_*total*_ of each sample in this group is calculated. The inset in Fig. 5(b) shows *R*_*total*_ versus *L* of both the single-layered *β*-W films (black rectangles) and the multilayered *β*-W films (red circles). We can see that all these points show a linear relationship and that the differences of *R*_*total*_ between the single-layered *β*-W films and the multilayered ones are not significant. This points out that *R*_*W/W*_ will be small. It is determined as below.

*R*_*total*_ of the multilayered *β*-W films can be expressed as the following equation:

4a $${R}_{total}=L/k+{R}_{W/Si{O}_{2}/Si}+n{R}_{W/W}.$$

So, we have

4b $${\rm{\Delta }}R={R}_{total}-{R}_{W/Si{O}_{2}/Si}=L/k+n{R}_{W/W}.$$

${R}_{W/Si{O}_{2}/Si}$ determined in the above section can be used here for data processing since the film synthesis follows the exactly same condition. And then we have

4c $$\frac{{\rm{\Delta }}R}{n}=\frac{L}{n}\frac{1}{k}+{R}_{W/W}.$$

When plotting Δ*R*/*n* versus *L*/*n*, these points also show a linear relationship as shown in Fig. 5(b). From equation (4c) we can see that the slope of the linear relation is related to the reciprocal of *k* and the intercept is related to *R*_*W/W*_. *k* determined here (2.05 ± 0.36 Wm^−1^K^−1^) is a little higher than (but very close to) that of the single-layered *β*-W films in the first group (1.98 ± 0.06 Wm^−1^K^−1^). Considering the uncertainties of the experiment and data processing, we conclude that the samples from the two groups have negligible structure difference during sample preparation. From this linear fitting, *R*_*W/W*_ is determined at (3.57 ± 2.67) × 10^−9^ Km^2^W^−1^. This *R*_*W/W*_ investigation serves as a comparison base for the *R*_*W/G*_ study that will be described in the next section. At this point, we can conclude that the *β*-W/*β*-W interface thermal conductance (*G*_*W/W*_) has an average of about 280 MW m^−2^K^−1^.

The electrical conductivity and resistivity in the in-plane direction of these multilayered *β*-W films has also been measured by using the four-probe method. Although there are some variations in several samples, the electrical conductivity and resistivity of the samples in this group have no significant difference with those of the first group. Considering the large thickness difference between the single-layered films and the multi-layered ones, the previous assumption that the size-effect or surface-scattering play a tiny role in the very high resistivity has also been proved. The electron thermal conductivity in the in-plane direction is also calculated using the WF law, which is larger but still comparable to that in the cross-plane direction. The calculation results are detailed in the Supplementary materials (see Table 3 in S4).

### Thermal Conductance between W and Graphene

**Figure 6:**
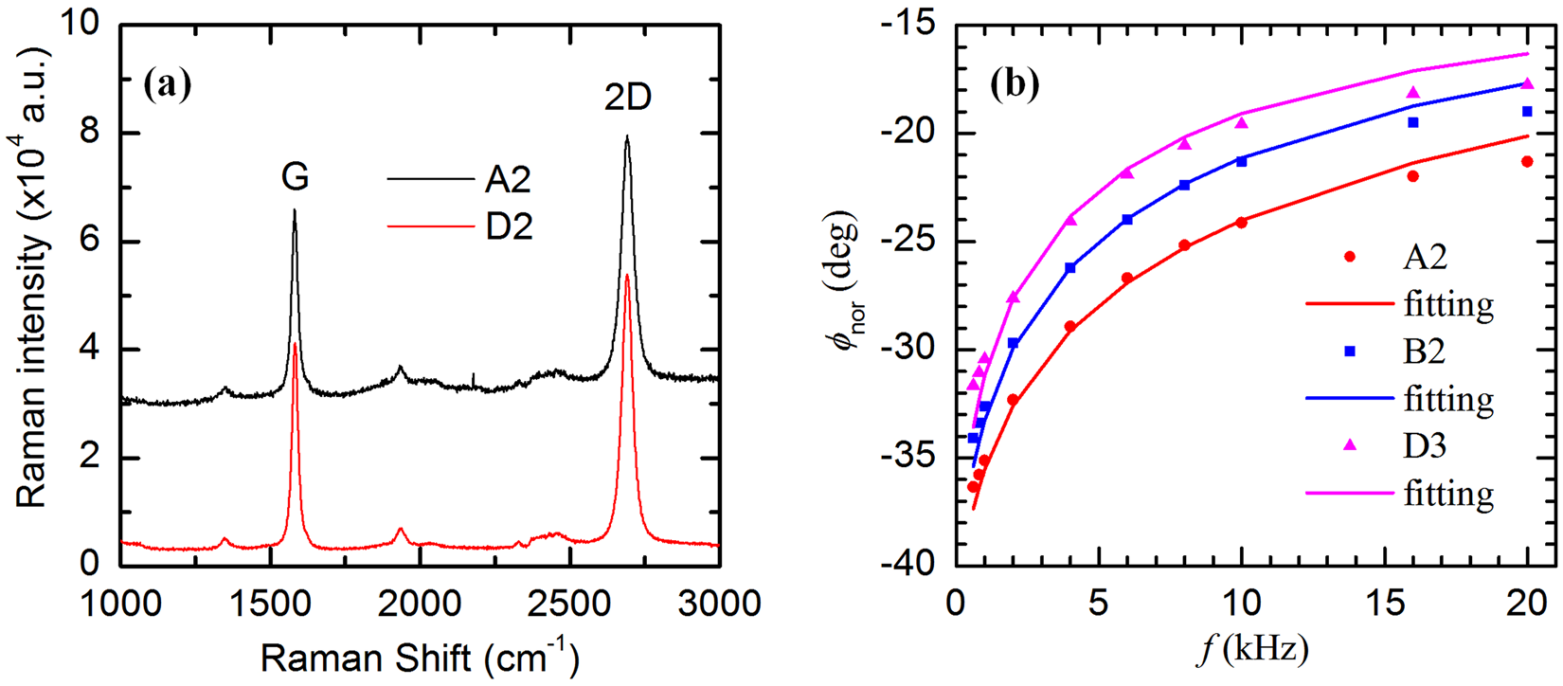
(**a**) Raman spectra of the graphene on the surface of two of our samples (A2 and D2). The G peak at 1583 cm^−1^ and 2D peak at 2690 cm^−1^ can be seen clearly. (**b**) Phase shift fitting for three of our samples (A2: 6 layers *β*-W films one-by-one sandwiched with 5 graphene layers and have a total thickness of 90 nm; B2: 6 layers *β*-W films one-by-one sandwiched with 5 graphene layers and have a total thickness of 180 nm; D3: 8 layers of *β*-W one-by-one sandwiched with 7 graphene layers and have a total thickness of 320 nm).

The samples in this group have the same thicknesses as those in the second group, respectively. Compared with samples in the second group, the difference is that we have graphene layers sandwiched between tungsten sublayers [see Fig. 1(a)]. Figure 6(a) shows the Raman spectra of the graphene layers in two of our samples, from which the G peak and 2D peak can be seen clearly. According to Graf and Molitor’s recent work^53^, the graphene used here are 1~2 layers graphene. Details of the layer number characterization are shown in the Methods section. The experimental setup of the PT technique is the same as those of the first two groups. What we can get is *R*_*total*_ induced by the *β*-W films, *R*_*W/G*_, and ${R}_{W/Si{O}_{2}/Si}$. Figure 6(b) shows the fitting result of several samples from this group. We can see the theoretical phase shifts match the experimental data very well. For single-layered graphene, the graphene-induced thermal resistance is from the two *β*-W/G interfaces. For there are two-layered graphene, thermal resistance across the Graphene/Graphene interface (*R*_*G/G*_) can be estimated as *R*_*G/G*_ = *l/k*_*G*_ with *l* the distance between graphene layers (0.335 nm) and *k*_*G*_ the cross-plane thermal conductivity of graphene (5.7 Wm^−1^K^−1^)^54^. Accordingly, *R*_*G/G*_ is about 5.9 × 10^−11^ Km^2^W^−1^ which is much smaller than *R*_*total*_. Thus, the intrinsic thermal resistance of the graphene layers can be neglected in this experiment. With *k* of *β*-W films taken as 1.69~2.41 Wm^−1^K^−1^ and ${R}_{W/Si{O}_{2}/Si}$ taken as (1.43 ± 0.2) × 10^−7^ Km^2^W^−1^, *R*_*W/G*_ of all the samples in this group can be calculated by using the following equation:

5 $${R}_{total}=L/k+{R}_{W/Si{O}_{2}/Si}+2n{R}_{W/G}$$

The calculation results are shown in Table 1, from which we can see *R*_*W/G*_ varies from sample to sample and has a maximum value of 9.67×10^−9^ Km^2^W^−1^. The lower and upper limit uncertainties are also given in Table 1. These uncertainties show the maximum possible variations of the calculated *R*_*W/G*_ of each sample. We can see the uncertainties also vary from sample to sample and has a maximum value of 2.43 × 10^−9^ Km^2^W^−1^. These uncertainties are mainly attributed to the uncertainty of *k* of *β*-W films, which varies from 1.69 to 2.41 Wm^−1^K^−1^.

**Table 1:** Thermal resistance (10^−9^ Km^2^W^−1^) of β-W/G interfaces.

Sample	A1	A2	A3	B1	B2	B3	D1	D2	D3
***R***_***W/G***_	${1.05}_{-0.95}^{+0.97}$	${5.08}_{-0.8}^{+0.82}$	${3.82}_{-0.76}^{+0.78}$	$-{0.05}_{-1.78}^{+1.8}$	${8.12}_{-1.62}^{+1.64}$	${8.7}_{-1.56}^{+1.59}$	${5.67}_{-2.35}^{+2.43}$	${9.67}_{-2.19}^{+2.21}$	${6.23}_{-1.86}^{+2.14}$
***R***_***2W/G***_	${2.10}_{-1.9}^{+1.93}$	${11.6}_{-1.6}^{+1.64}$	${7.64}_{-1.53}^{+1.56}$	$-{0.1}_{-3.57}^{+3.6}$	${16.2}_{-3.24}^{+3.28}$	${17.4}_{-3.13}^{+3.17}$	${11.3}_{-4.7}^{+4.87}$	${19.3}_{-4.38}^{+4.42}$	${12.5}_{-3.71}^{+4.29}$
***R***_***TW/G***_	6.30	58.0	53.5	−0.30	81.2	122	34.0	96.7	87.2

Subscript “_G_” represents graphene layers. “*R*_*2W/G*_” represents thermal resistance induced by a single graphene layer while “*R*_*TW/G*_” represents thermal resistance induced by all graphene layers in the sample.

Note that, for each graphene layer, it has two W/G interfaces (next to the top and bottom *β*-W layers). In Table 1, *R*_*2W/G*_ represents thermal resistance of two W/G interfaces induced by each graphene layer. Also shown in Table 1 is the total thermal resistance (named as *R*_*TW/G*_) induced by all *β*-W/G interfaces in one sample. Most of the *R*_*TW/G*_ values are much larger than the experimental uncertainty (8~9 × 10^−9^ Km^2^W^−1^). This means the thermal resistance induced by the graphene layers is detectable in our experiment. It is clear that most *R*_*2W/G*_ is larger than the derived *R*_*W/W*_ in the last section, indicating the introduction of graphene layers indeed gives rise to a finite interface thermal resistance. Taking into consideration of the uncertainties, *R*_*W/G*_ is no larger than 11.9×10^−9^ Km^2^W^−1^. This value is comparable to the graphene/SiO_2_ interface resistance (5.6~12 × 10^−9^ Km^2^W^−1^) reported by Chen^9^ while is much smaller than that of the epitaxial graphene and SiC interface (5.3 × 10^−5^ Km^2^W^−1^) reported by Yue^55^. The corresponding thermal conductance (G_*W/G*_) is 84 MWm^−2^K^−1^ which is also at the high end of the graphene/SiO_2_ interface (20~110 MWm^−2^K^−1^) reported by Mak and Liu^10^ and that of the Au/Ti/graphene/SiO_2_ interface (about 25 MWm^−2^K^−1^) reported by Koh and Bae^11^. This *G*_*W/G*_ value is much larger than 4~5 MWm^−2^K^−1^ reported by Jagannadham on characterizing thermal transport properties of W/graphene/Cu structure^14^. However, in his measurement, the structure was annealed at high temperature where reaction took place between W and graphene which reduces the interface thermal conductance significantly. Recent work by Huang *et al*.^15^ reported measurements on thermal conductance of Al/transferred graphene (trG)/Cu and Al/grown graphene (grG)/Cu interfaces, which is a good representative of the metal/graphene/metal interfaces. The reported *G* of Al/trG/Cu interfaces is about 20 MWm^−2^K^−1^, 35% lower than that of the Al/grG/Cu interfaces (about 31 MWm^−2^K^−1^). This lower *G* of the Al/trG/Cu interfaces is attributed to the lower conformity of trG to Cu substrate, which is further confirmed by the increase of *G* after the annealing treatment. The different degree of conformity could also be a reason for the variations of our measurement results. Despite the variations, our calculated *G*_*2W/G*_ is no smaller than 42 MWm^−2^K^−1^, still larger than 31 MWm^−2^K^−1^ of the intrinsic value of their Al/G/Cu interface. The relatively large interface conductance of our *β*-W/G/*β*-W interfaces is due to the unique structure of the samples. One possible reason is that the graphene transfer and processing will inevitably introduce functional groups or defects to the graphene surface. These functional groups or defects may enhance the energy coupling between *β*-W films and graphene. Another reason may be the damage of graphene during the *β*-W sputtering progress, where additional channels of direct heat transport between the *β*-W films form and significantly enhance the thermal conductance of the interfaces. This damage enhanced phenomenon has been studied in the most recent work by Huang *et al*.^16^. In this work, they reported measurements of thermal conductance of Pd/trG/Pd interface with the top Pd prepared by either thermal evaporation or rf magnetron sputtering. The results shown that, *G* of the sample with the rf magnetron sputtering Pd is 300 MWm^−2^K^−1^ at room temperature, seven times larger than that with the thermal evaporation Pd (42 MWm^−2^K^−1^). This enhancement is attributed to the electronic heat transport via atomic scale pinholes formed in the graphene during sputtering process, which has also been proved by the AFM characterization. This can also help explain the variations and even negative value of *R*_*W/G*_ in Table 1 as the graphene transfer process is manual and the damage level from metal sputtering cannot be predicted.

The four-probe measurement is also performed on the samples in this group and the results are summarized in the Supplementary materials (see Table 4 in S4). The electrical resistivity varies from 270 to 351 µΩ·cm, a little larger than those of the first two groups due to the inserted graphene layers but still in the well-accepted range (150–350 µΩ·cm)^38^. *σ* and correspondingly in-plane electron-induced *k* are also calculated (see Table 4 in S4). *k* varies around 3 Wm^−1^K^−1^ which is also comparable to those of the single-layered and multilayered *β*-W samples detailed in Supplementary materials (see Tables 2 and 3 in S4).

## Conclusion

In this work, we have conducted systematic studies of the thermal resistance and conductance at the *β*-W/G interfaces. Single-layered, multilayered *β*-W films and multilayered *β*-W films sandwiched with graphene layers were deposited on the 100-nm-SiO_2_/Si substrate using the magnetron sputtering method. Using our differential technology, we are able to distinguish the thermal conductivity and the interface thermal resistance. The crystallite size was determined to be 11 nm from the (200) peak of *β−*W. The pole-figure XRD shows that the *β*(200) plane is along the in-plane direction of the films. Based on the G and 2D peaks from the Raman spectra, the graphene samples were determined to be 1~2 layers. The thermal conductivity of *β*-W films is very low (1.69~2.41 Wm^−1^K^−1^) compared with that of the bulk *α*-phase tungsten (174 Wm^−1^K^−1^). This low *k* is mainly due to strong electron scattering of defects. The *β*-W/*β*-W interface thermal resistance was determined at (3.57 ± 2.67) × 10^−9^ Km^2^W^−1^, indicating excellent bonding and energy coupling at the W/W interface. The *β*-W/G interface thermal resistance was also determined. For each *β*-W/G interface, *R*_*W/G*_ did vary from sample to sample, largely due to the inconsistency in sample preparation and unknown graphene structure damage and alternation. Taking into consideration of the uncertainties, the largest *R*_*W/G*_ is 11.9 × 10^−9^ Km^2^W^−1^, corresponding to a lower bound of thermal conductance of 84 MWm^−2^K^−1^. Compared with the up-to-date reported graphene interface thermal conductance, the *β*-W/G interface thermal conductance is at the high end. The cross-plane thermal conductivity of *β*-W is smaller but still comparable to that of in the in-plane direction derived from the four-probe measurements and Wiedemann-Franz law.

## Methods

### Sample Preparation

The *β*-W/G multilayered film is synthesized by depositing tungsten film and transferring monolayered graphene to the tungsten film. Monolayered graphene films are grown on 25-μm thick Cu foils (Alfa Aesar, item No. 46365) by chemical vapor deposition (CVD). The transferring process of graphene is as follows. First, a PMMA (polymethyl methacrylate) layer is spin-coated on one side of the Cu foils and the graphene on the other side of Cu is cleaned by oxygen plasma. Then, the Cu foil is etched by FeCl_3_ solution. The remained graphene/PMMA is floated on the surface of FeCl_3_ solution, and is then cleaned by DI-water. The cleaned graphene/PMMA is transferred onto the surface of the tungsten film which is produced in an ultra-high vacuum magnetron sputtering system (ULVAC, ACS-4000-C4) at room temperature. Finally, the PMMA layer is dissolved by acetone to form the contact between tungsten and graphene. Through repeating the above process, the tungsten-graphene multilayered film can be obtained. It is worth noting that in one tungsten-graphene cyclic multilayered film, the thickness of each layer of tungsten film is kept the same. For convenience, in the following discussion, at the present of graphene, we name the thickness of each layer of tungsten film (which is 15, 30, 40 nm) as ‘A’, ‘B’, ‘D’, respectively [see Fig. 1(a)]. For comparison study, we also prepare multilayered tungsten samples where each layer shares the thickness as that of the tungsten-graphene structure. While there is no graphene, each tungsten layer (which is also 15, 30, 40 nm thick) is named as ‘a‘, ‘b‘, ‘d‘, respectively [see Fig. 1(b)]. In addition, Aa, Bb and Dd is corresponding to those single-layered tungsten samples [see Fig. 1(c)]. In this work, A1, A2, A3 mean that the number of the cycles of tungsten-graphene system is 3, 5, 7. The lateral dimensions of all the samples are about 1 cm × 1 cm and the detailed information of thickness can be found in Supplementary materials (see S1). To mention that, during the sample preparation, the samples are kept in the chamber all through the several sputtering processes, which makes sure that no tungsten oxide forms between the tungsten sublayers. Even for the multi-layered tungsten/graphene films, where the samples are taken out for graphene transfer, tungsten oxide is not likely to form. As we know, tungsten belongs to inert metal which is very stable in normal environment. Anna Warren *et al*.^56^ had ever placed a 1-mm-thick tungsten film in an oxygenated environment and investigated the oxidation behavior of tungsten under different temperature. It is found that the tungsten oxide was less than 1 nm even though the sample was exposed to air and heated to 100 °C for hours. While during our sample preparation process, the samples are immediately sent back to the vacuum chamber for sputtering the next tungsten layer after the graphene transfer process. The XRD patterns [see Fig. 2(a)] also show that there is no tungsten oxide formed.

### Structure characterization

The structure of the tungsten films is explored through XRD to investigate phase, crystallite orientation and grain size. In bulk tungsten, stable structure of tungsten is mainly body-centered-cubic (bcc) *α*-W. However, a metastable form of *β*-W can also appear in thin films^30,47,57,58^. The black line in Fig. 2(a) shows the XRD patterns of the investigated tungsten films in a 2θ range. We can see the strongest *β*(200) diffraction peaks of tungsten, which means the sample is mostly *β*-W with no other crystalline or amorphous and the preferred crystalline orientation is the *β*(200) direction^39,59^. The columnar structure shown in Fig. 4 also proves this based on the relations of microstructure and phase composition of W detailed in Shen’s work^60^. The *β*(210), *β*(312) and *β*(400) peaks can also be found in the black line but are much weaker than *β*(200). The blue line in Fig. 2(a) shows the XRD patterns of W films coated on a stainless steel substrate by gas tunnel type plasma spraying which is used for comparison purpose^61^. We can see that the (110) diffraction peaks of *α*-W is the strongest, followed by the *α*(211) peaks and then the *α*(200) peaks. The large difference of these two XRD patterns reveals different phase of W. The crystallites or grain size of the tungsten films is also estimated using the Scherrer equation^62^ from the *β*(200) peak in the diffractogram, which is determined at 11 nm. Figure 2(b) shows the schematic of the pole figure XRD measurement. This characterization is intended to determine the crystalline orientation in our samples. During this measurement, the diffraction angle (2θ) is fixed and the diffracted intensity is collected by varying two geometrical parameters, the *α* angle (tilt angle from sample surface normal direction) and the *β* angle (rotation angle around the sample surface normal direction). Figure 2(c) shows the schematic of the definition of *α* and *β* angle. Figure 2(d) is the pole-figure of the (200) plane of this tungsten film, from which we can conclude that the (200) plane is parallel to the surface of the sample as the maximum pole is located in the center of the pole figure where *α* = 0°.

AFM characterization is performed on the transferred graphene and the image shows kind of wrinkles in the graphene sheet while PMMA residues hardly appear. The AFM image is detailed in the Supplementary materials (see Fig. 3 in S5). The structure of graphene layers is also characterized by using Raman spectroscopy. In this experiment, the Raman spectra are collected by a confocal Raman spectrometer with a spectral resolution of 1.05~1.99 cm^−1^. A 20× objective lens is used, with an integration time of 10 s and a laser spot size of 2.01 μm. The laser energy reaching the surface of the samples is 44.7 mW. Figure 6(a) shows the Raman spectra of graphene on two of our samples. The G peak and 2D peak can be seen clearly. Based on the work of Graf and Molitor^53^, the ratio of the integrated intensities of the G and 2D peaks (named *I*_*G/2D*_) can be used as a parameter to determine the number of layers of the graphene flake. From Fig. 6(a), the G and 2D peaks for the two samples are fitted and the integrated intensities are calculated separately. The ratio of integrated intensities of the G and 2D peaks (*I*_*G/2D*_) of the two samples are calculated at 0.38 and 0.42, respectively which means the graphene used in our experiment is 1~2 layered graphene^53^.

## Electronic supplementary material

Supplementary information

## References

[1] Geim AK, Novoselov KS (2007). The rise of graphene. Nat. Mater..

[2] Balandin AA (2008). Superior thermal conductivity of single-layer graphene. Nano Lett..

[3] Xin GQ (2015). Highly thermally conductive and mechanically strong graphene fibers. Sci.

[4] Novoselov KS (2004). Electric field effect in atomically thin carbon films. Sci..

[5] Zhou Q, Zheng JL, Onishi S, Crommie MF, Zettl AK (2015). Graphene electrostatic microphone and ultrasonic radio. P. Natl. Acad. Sci. USA..

[6] Han MY, Ozyilmaz B, Zhang YB, Kim P (2007). Energy band-gap engineering of graphene nanoribbons. Phys. Rev. Lett..

[7] Chen ZH, Lin YM, Rooks MJ, Avouris P (2007). Graphene nano-ribbon electronics. Physica. E..

[8] Freitag M (2009). Energy Dissipation in Graphene Field-Effect Transistors. Nano Lett..

[9] Chen Z, Jang W, Bao W, Lau CN, Dames C (2009). Thermal contact resistance between graphene and silicon dioxide. Appl. Phys. Lett..

[10] Mak KF, Lui CH, Heinz TF (2010). Measurement of the thermal conductance of the graphene/SiO2 interface. Appl. Phys. Lett..

[11] Koh YK, Bae MH, Cahill DG, Pop E (2010). Heat Conduction across Monolayer and Few-Layer Graphenes. Nano Lett..

[12] Guzman, P. A. V. *et al*. Cross Plane Thermal Conductance of Graphene-Metal Interfaces. Ieee Intersociety Conference on Thermal and Thermomechanical Phenomena in Electronic Systems 1385–1389 (2014).

[13] Hopkins PE (2012). Manipulating Thermal Conductance at Metal-Graphene Contacts via Chemical Functionalization. Nano Lett..

[14] Jagannadham K (2014). Effect of interfacial interactions on the thermal conductivity and interfacial thermal conductance in tungsten-graphene layered structure. J. Vac. Sci. Technol. A.

[15] Huang B, Koh YK (2016). Improved topological conformity enhances heat conduction across metal contacts on transferred graphene. Carbon.

[16] Huang, B. & Koh, Y. K. Negligible electronic contribution to heat transfer across intrinsic metal/graphene interfaces. *Adv*. *Mater*. *Interfaces* **1700559** (2017).

[17] Gao Y (2011). Radiation tolerance of Cu/W multilayered nanocomposites. J. Nucl. Mater..

[18] Pentecoste L (2016). Low Energy and low fluence helium implantations in tungsten: Molecular dynamics simulations and experiments. J. Nucl. Mater..

[19] Hasegawa A, Fukuda M, Yabuuchi K, Nogami S (2016). Neutron irradiation effects on the microstructural development of tungsten and tungsten alloys. J. Nucl. Mater..

[20] Bunch JS (2008). Impermeable atomic membranes from graphene sheets. Nano Lett..

[21] Kim Y (2016). Radiation resistant vanadium-graphene nanolayered composite. Scientific reports.

[22] Gibson JW, Hein RA (1964). Superconductivity of Tungsten. Phys. Rev. Lett..

[23] Bond WL (1965). Superconductivity in Films of Beta Tungsten and Other Transition Metals. Phys. Rev. Lett..

[24] Basavaiah S, Pollack SR (1968). Superconductivity in Evaporated Tungsten Films. Appl. Phys. Lett..

[25] Tang CC, Hess DW (1984). Plasma-Enhanced Chemical Vapor-Deposition of Beta-Tungsten, a Metastable Phase. Appl. Phys. Lett..

[26] Hartmann H, Ebert F, Bretschneider O (1931). Elektrolysen in Phosphatschmelzen. I. Die elektrolytische Gewinnung von α-und β-Wolfram. Z. Anorg. Allg. Chem..

[27] Fink CG, Ma CC (1943). Pure Tungsten Direct from Ore I. Electrolytic Tungsten from Fused Borax and Fused Phosphate Baths. Transactions of The Electrochemical Society.

[28] Rossnagel SM, Noyan IC, Cabral C (2002). Phase transformation of thin sputter-deposited tungsten films at room temperature. J. Vac. Sci. Technol., B.

[29] Choi D (2011). Phase, grain structure, stress, and resistivity of sputter-deposited tungsten films. J. Vac. Sci. Technol., A.

[30] OKeefe MJ, Grant JT (1996). Phase transformation of sputter deposited tungsten thin films with A-15 structure. J. Appl. Phys..

[31] Pai CF (2012). Spin transfer torque devices utilizing the giant spin Hall effect of tungsten. Appl. Phys. Lett..

[32] Hao Q, Chen WZ, Xiao G (2015). Beta (beta) tungsten thin films: Structure, electron transport, and giant spin Hall effect. Appl. Phys. Lett..

[33] Girault B (2013). Controlled nanostructuration of polycrystalline tungsten thin films. J. Appl. Phys..

[34] Wang XW, Zhong ZR, Xu J (2005). Noncontact thermal characterization of multiwall carbon nanotubes. J. Appl. Phys..

[35] Chen XW, He YP, Zhao YP, Wang XW (2010). Thermophysical properties of hydrogenated vanadium-doped magnesium porous nanostructures. Nanot.

[36] Wang T (2008). Effect of zirconium(IV) propoxide concentration on the thermophysical properties of hybrid organic-inorganic films. J. Appl. Phys..

[37] Kittel, C. *Introduction to solid state physics*. 8 edn (Wiley, 2005).

[38] Petroff P, Sheng TT, Sinha AK, Rozgonyi GA, Alexander FB (1973). Microstructure, Growth, Resistivity, and Stresses in Thin Tungsten Films Deposited by Rf Sputtering. J. Appl. Phys..

[39] Lee JS, Cho J, You CY (2016). Growth and characterization of alpha and beta-phase tungsten films on various substrates. J. Vac. Sci. Technol. A.

[40] Bowler N, Huang YQ (2005). Model-based characterization of homogeneous metal plates by four-point alternating current potential drop measurements. Ieee. T. Magn..

[41] Bowler N, Huang YQ (2005). Electrical conductivity measurement of metal plates using broadband eddy-current and four-point methods. Meas. Sci. Technol..

[42] Smits F (1958). Measurement of sheet resistivities with the four‐point probe. BELL. SYST. TECH. J..

[43] Fuchs K (1938). The conductivity of thin metallic films according to the electron theory of metals. Proceedings of the Cambridge Philosophical Society.

[44] Sondheimer EH (1952). The Mean Free Path of Electrons in Metals. AdPhy.

[45] Mayadas AF, Shatzkes M, Janak JF (1969). Electrical Resistivity Model for Polycrystalline Films - Case of Specular Reflection at External Surfaces. Appl. Phys. Lett..

[46] Mayadas AF, Shatzkes M (1970). Electrical-Resistivity Model for Polycrystalline Films - Case of Arbitrary Reflection at External Surfaces. Phys. Rev. B.

[47] Shen YG, Mai YW (2001). Structure and properties of stacking faulted A15 tungsten thin films. J. Mater. Sci..

[48] Yoneoka S (2012). Electrical and thermal conduction in atomic layer deposition nanobridges down to 7 nm thickness. Nano Lett..

[49] Zhang Q, Cao B, Zhang X, Fujii M, Takahashi K (2006). Influence of grain boundary scattering on the electrical and thermal conductivities of polycrystalline gold nanofilms. PhRvB.

[50] Wang H-D, Liu J-H, Zhang X, Guo Z-Y, Takahashi K (2011). Experimental study on the influences of grain boundary scattering on the charge and heat transport in gold and platinum nanofilms. Heat Mass Transfer..

[51] Ou M (2008). Electrical and thermal transport in single nickel nanowire. Appl. Phys. Lett..

[52] Lin H, Xu S, Wang X, Mei N (2013). Thermal and Electrical Conduction in Ultrathin Metallic Films: 7 nm down to Sub‐Nanometer Thickness. Small.

[53] Graf D (2007). Spatially resolved raman spectroscopy of single- and few-layer graphene. Nano Lett..

[54] Ho CY, Powell RW, Liley PE (1972). Thermal conductivity of the elements. J. Phys. Chem. Ref. Data.

[55] Yue YN, Zhang JC, Wang XW (2011). Micro/Nanoscale Spatial Resolution Temperature Probing for the Interfacial Thermal Characterization of Epitaxial Graphene on 4H-SiC. Small.

[56] Warren A, Nylund A, Olefjord I (1996). Oxidation of tungsten and tungsten carbide in dry and humid atmospheres. International Journal of Refractory Metals & Hard Materials.

[57] Radic N (2004). Sputter-deposited amorphous-like tungsten. Surf. Coat. Tech..

[58] Noyan IC, Shaw TM, Goldsmith CC (1997). Inhomogeneous strain states in sputter deposited tungsten thin films. J. Appl. Phys..

[59] Djerdj I, Tonejc AM, Tonejc A, Radic N (2005). XRD line profile analysis of tungsten thin films. Vacuu.

[60] Shen YG (2000). Residual stress, microstructure, and structure of tungsten thin films deposited by magnetron sputtering. J. Appl. Phys..

[61] Kobayashi A, Puric J (2009). Microstructure and hardness of tungsten coating for high heat resistant material produced by means of gas tunnel type plasma spraying. Trans. JWRI.

[62] Patterson AL (1939). The Scherrer formula for x-ray particle size determination. PhRv.

