# Supplementary material for: Interface Energy Coupling between β-tungsten Nanofilm and Few-layered Graphene
Source: Sci Rep. 2017 Sep 22;7:12213. doi: 10.1038/s41598-017-12389-1 (PMC5610176; doi:10.1038/s41598-017-12389-1)
Supplement: Supplementary file 1 — Supplementary information [file 41598_2017_12389_MOESM1_ESM.pdf]

Interface Energy Coupling between β-tungsten Nanofilm and Few-layered Graphene

Meng Han[a], Pengyu Yuan[a], Jing Liu[a], Shuyao Si[b], Xiaolong Zhao[b], Yanan Yue[c], Xinwei Wang[a,*], Xiangheng Xiao[b,*]

[a] 2010 Black Engineering Building, Department of Mechanical Engineering

Iowa State University, Ames, IA, 50011

[b] Department of Physics and Key Laboratory of Artificial Micro- and Nano-structures of Ministry of Education, Hubei Nuclear Solid Physics Key Laboratory and Center for Ion Beam Application, Wuhan University, Wuhan 430072, P. R. China

[c] School of Power and Mechanical Engineering, Wuhan University, Wuhan, Hubei 430072, P. R. China

[*] Corresponding authors. XW: xwang3@iastate.edu; XX: xxh@whu.edu.cn

S1. Structure information of the samples prepared and measured in the present work

Table 1. Index and geometries of all samples

Single W film thickness in the multilayered sample	Multilayered W/Graphene film	Multilayered W film and the layer number	Single-layered W film and the thickness
15 nm	A1: 3×[W+G]+W	a1 (4)	Aa1 (74 nm)
	A2: 5×[W+G]+W	a2 (6)	Aa2 (110 nm)
	A3: 7×[W+G]+W	a3 (8)	Aa3 (138 nm)
30 nm	B1: 3×[W+G]+W	b1 (4)	Bb1 (128 nm)
	B2: 5×[W+G]+W	b2 (6)	Bb2 (190 nm)
	B3: 7×[W+G]+W	b3 (8)	Bb3 (247 nm)
40 nm	D1: 3×[W+G]+W	d1 (4)	Dd1 (167 nm)
	D2: 5×[W+G]+W	d2 (6)	Dd2 (252 nm)
	D3: 7×[W+G]+W	d3 (8)	Dd3 (344 nm)

"7×[W+G]" means 7 cycles of "tungsten+graphene" layer.

S2. Experiment setup and the physical model

The noncontact PT technique[1-3] is used to characterize the thermal transport properties of a multilayer structure in the cross-plane direction. A modulated laser beam is used to irradiate the film surface which serves as a heat source and leads to a periodical temperature variation at the film surface. This temperature variation is strongly affected by the thermal conduction of the sample and is sensed by detecting the surface thermal radiation. As the thermal radiation is from the sample surface, we believe the detection can accurately reflect the real temperature variations of the sample surface. The phase shift of radiation to the laser beam is used to characterize the interface energy coupling properties of the samples.

Figure 1 (a) shows the experimental setup and how it is operated. A continuous infrared laser is modulated by a function generator and then is directed and focused on the sample. The sample is heated to different temperatures by the laser beam under different modulation frequencies. In this experiment, the modulated laser beam is 600 mW, which assures sufficiently high radiation signal from the surface while prevents high temperature rise that may damage the sample or significantly change the sample's thermophysical properties. The laser beam has a Gaussian distribution in space and the spot size is 0.7×1.4 mm in our experiment. As the dimension of the laser spot is much larger than the thermal diffusion length in the lateral direction of the sample, the Gaussian distribution has negligible influence on the measured phase shift signal. The thermal radiation signal is directed to an infrared detector which has a Ge window in front to filter out the reflection signal of the laser beam and only allows the thermal radiation signal to pass. The radiation signals are then pre-amplified and measured by a lock-in amplifier and the phase shift within a large laser frequency range is obtained for further data processing.

In this experiment, the modulation frequencies of the laser beam range from 600 Hz to 20 kHz, under which the thermal diffusion depth within one period heating is much smaller than the dimensions of the heating spot. Thus, the thermal transport process induced by the laser beam can be simplified to a one-dimensional cross-plane heat transfer model. Figure 1 (b) shows the cross-sectional view of the one-dimensional multilayer model. Layers from 1 to N are the sample layers between the substrate (layer 0) and the air (layer N+1). The governing equation for a multilayer one-dimensional thermal conduction problem in layer i can be expressed as[4]:

$$\frac{\partial^2\theta_i}{\partial x^2}=\frac{1}{\alpha_i}\frac{\partial\theta_i}{\partial t}-\frac{\beta_i I_0}{2k_i}\exp\left(\sum_{m=i+1}^{N}-\beta_m L_m\right)\times e^{\beta_i(x-l_i)}\left(1+e^{j\omega t}\right). \tag{1}$$

Here $L_i=l_i-l_{i-1}$ is the thickness of layer i. Other thermophysical properties of layer i are thermal conductivity k_i, specific heat $c_{p,i}$ and optical absorption coefficient β_i. $\theta_i=T_i-T_{amb}$ is the temperature rise of layer i while T_{amb} is the ambient temperature. ω is the angular frequency ($2\pi f$). The solution to equation 1 consists of a transient component $\theta_{i,t}$, a steady DC component $\overline{\theta}_{i,s}$ and a steady AC component $\tilde{\theta}_{i,s}$. In this experiment, only the steady AC component $\tilde{\theta}_{i,s}$ is measured. The general solution to this part follow the below form[4]:

$$\tilde{\theta}_{i,s}=\left[\mathrm{A}_i e^{\sigma_i(x-l_i)}+\mathrm{B}_i e^{-\sigma_i(x-l_i)}-\mathrm{E}_i e^{\beta_i(x-l_i)}\right]e^{j\omega t}. \tag{2}$$

In the above equation, $E_i=G_i\left(\beta_i^2-\sigma_i^2\right)$ with $G_i=\frac{\beta_i I_0}{2k_i}\exp\left(-\sum_{m=i+1}^{N}\beta_m L_m\right)$ for $i<N$, $G_N=\beta_N I_0/2k_N$, and $G_{N+1}=0$. σ_i is defined as $(1+j)\cdot a_i$ with $j=\sqrt{-1}$.

The coefficient A_i and B_i can be determined by using the interfacial conditions at $x=l_i$ as:

$$\begin{bmatrix}\mathrm{A}_i\\ \mathrm{B}_i\end{bmatrix}=U_i\begin{bmatrix}\mathrm{A}_{i+1}\\ \mathrm{B}_{i+1}\end{bmatrix}+V_i\begin{bmatrix}E_i\\ E_{i+1}\end{bmatrix}, \tag{3}$$

where U_i is the interfacial transmission matrix of heat and V_i is the absorption matrix of light from layer $i+1$ to i and they can be expressed as:

$$U_i = \frac{1}{2}\begin{bmatrix} u_{11,i} & u_{12,i} \\ u_{21,i} & u_{22,i} \end{bmatrix}; V_i = \frac{1}{2}\begin{bmatrix} v_{11,i} & v_{12,i} \\ v_{21,i} & v_{22,i} \end{bmatrix}. \quad (4)$$

$$u_{1n,i} = \left(1 \pm k_{i+1}\sigma_{i+1}/k_i\sigma_i \mp k_{i+1}\sigma_{i+1}R_{i,i+1}\right) \times \exp\left[\mp\sigma_{i+1}\left(l_{i+1} - l_i\right)\right], n = 1,2, \quad (5a)$$

$$u_{2n,i} = \left(1 \mp k_{i+1}\sigma_{i+1}/k_i\sigma_i \mp k_{i+1}\sigma_{i+1}R_{i,i+1}\right) \times \exp\left[\mp\sigma_{i+1}\left(l_{i+1} - l_i\right)\right], n = 1,2, \quad (5b)$$

$$v_{n1,i} = 1 \mp \beta_i/\sigma_i, n = 1,2 \;, \quad (5c)$$

and $$v_{n2,i} = \left(-1 \mp k_{i+1}\beta_{i+1}/k_i\sigma_i \mp k_{i+1}\beta_{i+1}R_{i,i+1}\right) \times \exp\left[-\beta_{i+1}\left(l_{i+1} - l_i\right)\right], n = 1,2. \quad (5d)$$

$R_{i,i+1}$ is the thermal contact resistance between layer i and i+1. Details of the parameters are provided in Ref. 4.

From the above solution, the phase shift between thermal radiation of the sample surface and the irradiating laser beam can be derived. Then the least square method is used to determine unknown thermophysical properties of the sample. Notice that, the optical absorption depth (τ_{opt}) is a key parameter in the PT measurement and the fitting process. In this work, τ_{opt} is taken as 22.8 nm for the β-W films. This value is larger than the thickness of top layers of several samples. The second or third layers may absorb laser energy. This has also been taken into full consideration by given τ_{opt} of these layers in the fitting program.

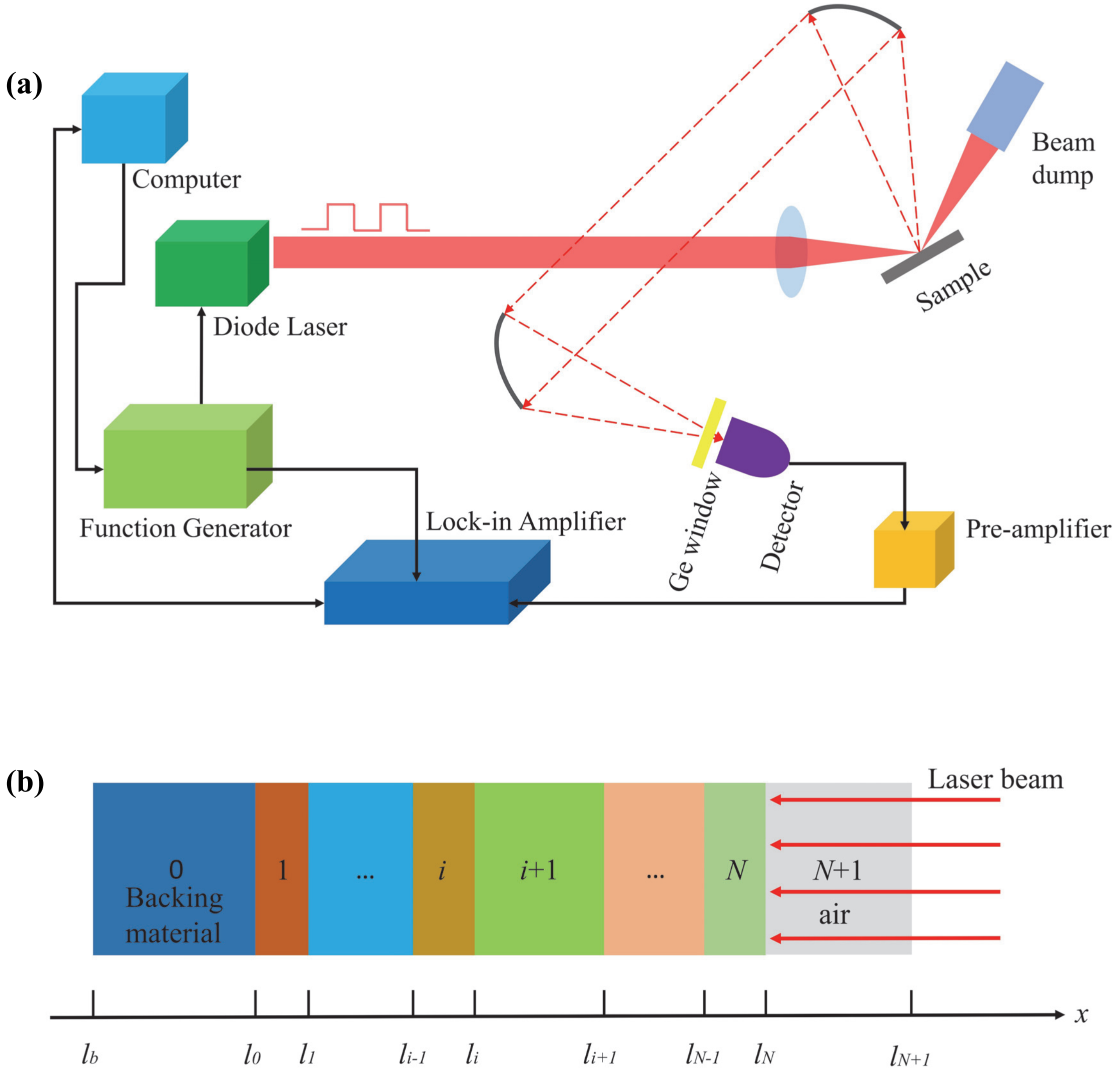

Fig. 1 (a) Schematic of the photothermal experimental setup. (b) Schematic of an N-layer sample to show a multilayered model used in the data processing.

S3. Schematic and experiment setup of the four-probe method

Figure 2(a) shows the schematic of the four-probe method. The two out probes (probes 1 and 4) are fed with a DC current while the voltage is measured from the two inner probes (probes 2 and 3). Figure 2(b) shows the experiment setup of the four-probe method in our lab. The four probes are fixed on the 3D microstage which makes the contact between probes and the sample much flexible. The orange wires are connected with a DC current source and multimeter while the copper piece below the four pins represents the samples that are measured in our experiment.

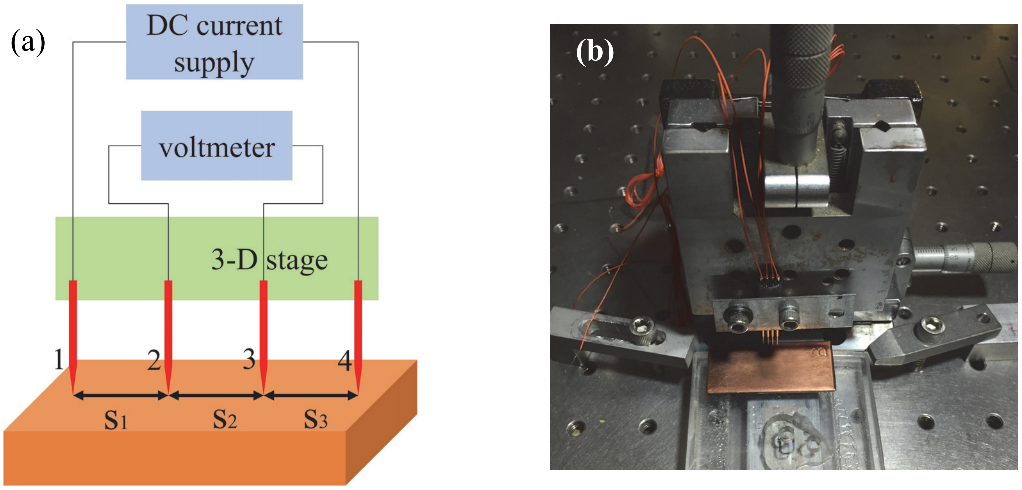

Fig. 2 (a) Schematic of the four-probe method for measuring the in-plane electrical conductivity of films. (b) Experimental setup of the four-probe measurement in our lab.

S4. Measurement results through the four-probe method

Table 2. In-plane electrical conductivity and thermal conductivity of single-layered *β*-W

Samples	V/I (A)	F_1	σ ($\times 10^5$ $\Omega^{-1}m^{-1}$)	ρ ($\mu\Omega\cdot cm$)	k ($Wm^{-1}K^{-1}$)
Aa1	9.85	4.03	3.40	294	3.11
Aa2	5.79	4.05	3.87	258	3.53
Aa3	5.30	3.84	3.56	281	3.25
Bb1	5.77	3.94	3.44	291	3.14
Bb2	3.92	3.72	3.61	277	3.29
Bb3	3.12	3.92	3.31	302	3.02
Dd1	4.59	3.91	3.33	300	3.04
Dd2	3.14	3.99	3.17	316	2.89
Dd3	2.47	2.83	3.56	281	3.25

Table 3. In-plane electrical conductivity and thermal conductivity of multilayered β-W

samples	V/I (A)	F_1	σ ($\times 10^5\ \Omega^{-1}m^{-1}$)	ρ ($\mu\Omega\cdot cm$)	k ($Wm^{-1}K^{-1}$)
a1	9.48	3.79	4.64	215	4.23
a2	4.66	4.05	5.89	167	5.38
a3	2.94	3.90	7.25	138	6.61
b1	6.31	3.91	3.38	296	3.08
b2	4.23	3.75	3.50	286	3.19
b3	2.67	3.83	4.07	246	3.71
d1	5.23	3.29	3.63	275	3.31
d2	3.15	3.60	3.67	272	3.34
d3	2.27	3.72	3.71	270	3.38

Table 4. In-plane electrical conductivity and thermal conductivity of multilayered *β*-W/G

samples	V/I (A)	F_1	σ ($\times 10^5$ $\Omega^{-1}m^{-1}$)	ρ ($\mu\Omega\cdot cm$)	k ($Wm^{-1}K^{-1}$)
A1	12.9	4.00	3.24	309	2.95
A2	7.53	3.98	3.70	270	3.38
A3	5.90	4.05	3.49	287	3.18
B1	6.17	3.85	3.51	285	3.20
B2	4.24	3.86	3.40	294	3.10
B3	3.36	4.02	3.09	324	2.82
D1	4.96	3.92	3.22	311	2.93
D2	3.69	3.96	2.85	351	2.60
D3	2.79	3.91	2.86	350	2.61

S5. Atomic-force microscopy (AFM) characterization of the transferred graphene

Figure 3 shows the AFM image of the transferred graphene on β-W film from which we can see several winkles appear in the graphene sheet.

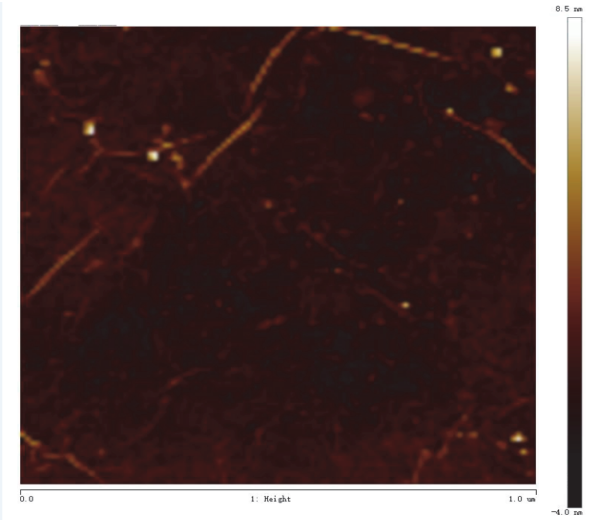

Fig. 3 AFM image of the transferred graphene on β-W film

References

1 Wang, X. W., Zhong, Z. R. & Xu, J. Noncontact thermal characterization of multiwall carbon nanotubes. *J. Appl. Phys.* **97**, 064302, (2005).

2 Chen, X. W., He, Y. P., Zhao, Y. P. & Wang, X. W. Thermophysical properties of hydrogenated vanadium-doped magnesium porous nanostructures. *Nanot* **21**, 055707, (2010).

3 Wang, T. *et al.* Effect of zirconium(IV) propoxide concentration on the thermophysical properties of hybrid organic-inorganic films. *J. Appl. Phys.* **104**, 013528, (2008).

4 Hu, H. P., Wang, X. W. & Xu, X. F. Generalized theory of the photoacoustic effect in a multilayer material. *J. Appl. Phys.* **86**, 3953-3958, (1999).
